# Does the evidence support the use of operating room shoe covers to prevent surgical site infections? A scoping review

**DOI:** 10.1017/ash.2026.10311

**Published:** 2026-02-24

**Authors:** Qi Rui Soh, Hadrien Moffroid, Edwina Eaton, Nicola Isles, Caroline Marshall, Brigid M. Gillespie, Ben Dunne

**Affiliations:** 1 Department of Surgery, Royal Melbourne Hospital, Melbourne, Victoria, Australia; 2 Department of Infectious Diseases, University of Melbourne, at the Peter Doherty Institute for Infection and Immunity, Melbourne, Victoria, Australia; 3 Australian College of Perioperative Nurses (ACORN), Australia; 4 Australian College for Infection Prevention and Control (ACIPC), Australia; 5 Infection Prevention and Surveillance Service, The Royal Melbourne Hospital, Melbourne, Victoria, Australia; 6 School of Nursing & Midwifery, Griffith University, Queensland, Australia

## Abstract

**Objective:**

To determine whether operating room (OR) shoe covers prevent surgical site infections (SSIs) and to assess their environmental and clinical impact.

**Design:**

Scoping review.

**Setting:**

Hospital operating room environments in international healthcare systems.

**Methods:**

We searched Emcare, Embase, MEDLINE, and SCOPUS for studies examining shoe covers and outcomes related to bacterial contamination or SSIs. Data were synthesized descriptively.

**Results:**

Six studies met inclusion criteria. Evidence was mixed regarding bacterial contamination: some showed fewer colony forming units with shoe covers, while others found no effect or even higher contamination. Only one study assessed clinical outcomes, reporting fewer SSIs following reduced use of disposable PPE (including shoe covers). No study demonstrated a direct SSI reduction from shoe covers alone.

**Conclusions:**

Evidence does not support OR shoe covers in preventing SSIs. Their use adds environmental burden through single-use plastics. More rigorous studies are needed to confirm these findings and guide sustainable infection prevention practices.

## Introduction

Healthcare systems contribute significantly to greenhouse gas emissions.^1^ Operating rooms (ORs) are an important target for initiatives to improve healthcare sustainability, given they are the source of a significant proportion of greenhouse gas emissions.^2^

Shoe covers worn by healthcare workers (HCWs) offer a prime example of a single-use product whose use could be reduced or eliminated as the theoretical basis for using them is not underpinned by the theory around the source of surgical site infections (SSIs). Reducing the unnecessary use of shoe covers may significantly reduce the carbon footprint of surgical care delivery, as most shoe covers are made from polyethylene or polypropylene, which have a carbon footprint of 1.8 to 2.3 kg CO_2_e (carbon dioxide equivalent) per kg^3^ and are not recyclable. Some evidence suggests that use of shoe covers risks contamination of HCW hands with pathogens when donning and doffing and could increase SSIs if appropriate hand hygiene is not performed.^7^

In the financial year 2023/24, a large tertiary hospital in Melbourne, Australia used 91,700 individual shoe covers at a cost of AUD $9536 (Internal Data) and an annual carbon footprint of between 1,008 kg and 1,283 kg CO_2_e (depending on emissions factors of 1.8 or 2.3, resulting in an emissions factor of between 11 and 14g CO2e per shoe cover).

### Intended purpose of operating room shoe covers

OR shoe covers are used by HCW while they are in the operating room complex. These shoe covers are normally worn over shoes and are intended to reduce contamination of the OR complex by pathogens and visible dirt brought in on shoes from the external environment. The use of other parts of surgical attire intended to prevent SSIs including head coverings, gloves, and surgical gowns, is described elsewhere.^4^

### Review of current guidelines

National and international guidelines on disposable surgical attire exist and include recommendations for the use of shoe covers in the OR.

As part of perioperative attire, the Australian College of Perioperative Nurses standards recommend the use of shoe covers when dedicated theater footwear is not being used.^5^ The Association of Perioperative Registered Nurses (AORN) guidelines recommends that fluid-resistant shoe covers or boots be worn when there is reasonable anticipation of gross contamination, such as from blood and/or bodily fluids.^6^ Similarly, the World Health Organization^7^ also published guidelines regarding using disposable shoe covers when a likelihood exists for bodily fluids to spill or leak.^8^ However, these guidelines do not specify a need for disposable shoe covers to be worn for the purpose of preventing SSIs.

For many years, it has been believed that, in the era of aseptic surgery, endogenous organisms are the predominant bacterial source of SSIs.^9,10^ Given that endogenous bacteria form the vast majority of SSI-causing organisms, this further undermines the evidence for shoe covers preventing SSIs, given the biological implausibility. However, there is limited contemporary consensus regarding the use of shoe covers as an infection prevention tool, as was highlighted in a review of current guidelines by the National Health Service in Scotland.^11^ This underscores the need for a more nuanced understanding of the literature surrounding the utility of shoe covers in preventing SSIs to better inform guidelines on their use. Given the sparse and inconclusive data, as well as the lack of controlled trials, a scoping review was deemed the most appropriate format to achieve this.

This review aims to describe the effectiveness of OR shoe covers in reducing surface contamination and SSIs, to explore the possibility of reducing their use in the OR. This effort exists within the larger context of reducing surgical waste and alleviating the environmental impact of surgical care delivery while maintaining the highest standards of care for our patients.

## Methods

The conduct and reporting of this review reflects the Preferred Reporting Items for Systematic reviews and Meta-Analyses (PRISMA) Statement.^12^

### Search strategy

A search strategy was developed and modified based on the subject headings of each database. The literature was sourced through searches of Emcare, Embase, MEDLINE, and SCOPUS databases (Supplementary Table 1). An example of the MEDLINE search strategy, including search terms and Boolean connectors, is shown here in Table 1.

**Table 1.** Example MEDLINE search strategy

Ovid MEDLINE(R) ALL <1946 to November 21, 2023>
1 Shoes/
2 shoe*.mp. [mp=title, book title, abstract, original title, name of substance word, subject heading word, floating sub-heading word, keyword heading word, organism supplementary concept word, protocol supplementary concept word, rare disease supplementary concept word, unique identifier, synonyms, population supplementary concept word, anatomy supplementary concept word]
3 1 or 2
4 Operating Rooms/
5 ((surgery or surgical) adj2 (room* or theater* or theater* or suite*)).mp. [mp=title, book title, abstract, original title, name of substance word, subject heading word, floating sub-heading word, keyword heading word, organism supplementary concept word, protocol supplementary concept word, rare disease supplementary concept word, unique identifier, synonyms, population supplementary concept word, anatomy supplementary concept word]
6 (operating adj2 (room* or theater* or theater* or suite*)).ti,ab,kf.
7 4 or 5 or 6
8 exp Infection Control/
9 (infect* adj2 (control* or prevent*)).ti,ab,kf.
10 8 or 9
11 3 and 7 and 10

### Inclusion and exclusion criteria

The inclusion criteria are as follows:

- Articles where primary research is reported using any type of design, either quantitative or qualitative, or both.
- Articles reporting on shoe covers in the context of infection control.
- Articles reporting on, or in context of, operating rooms, operating theaters, and surgical suites.
- Articles written in English.

The exclusion criteria are as follows:

- Articles reporting on shoes and/or shoe covers in a context of infection control in non-surgical settings, e.g., endoscopy units/ICU.
- Protocols, editorials, commentaries, and letters.
- Articles from before 1980.

### Screening and data extraction

**Figure 1.**
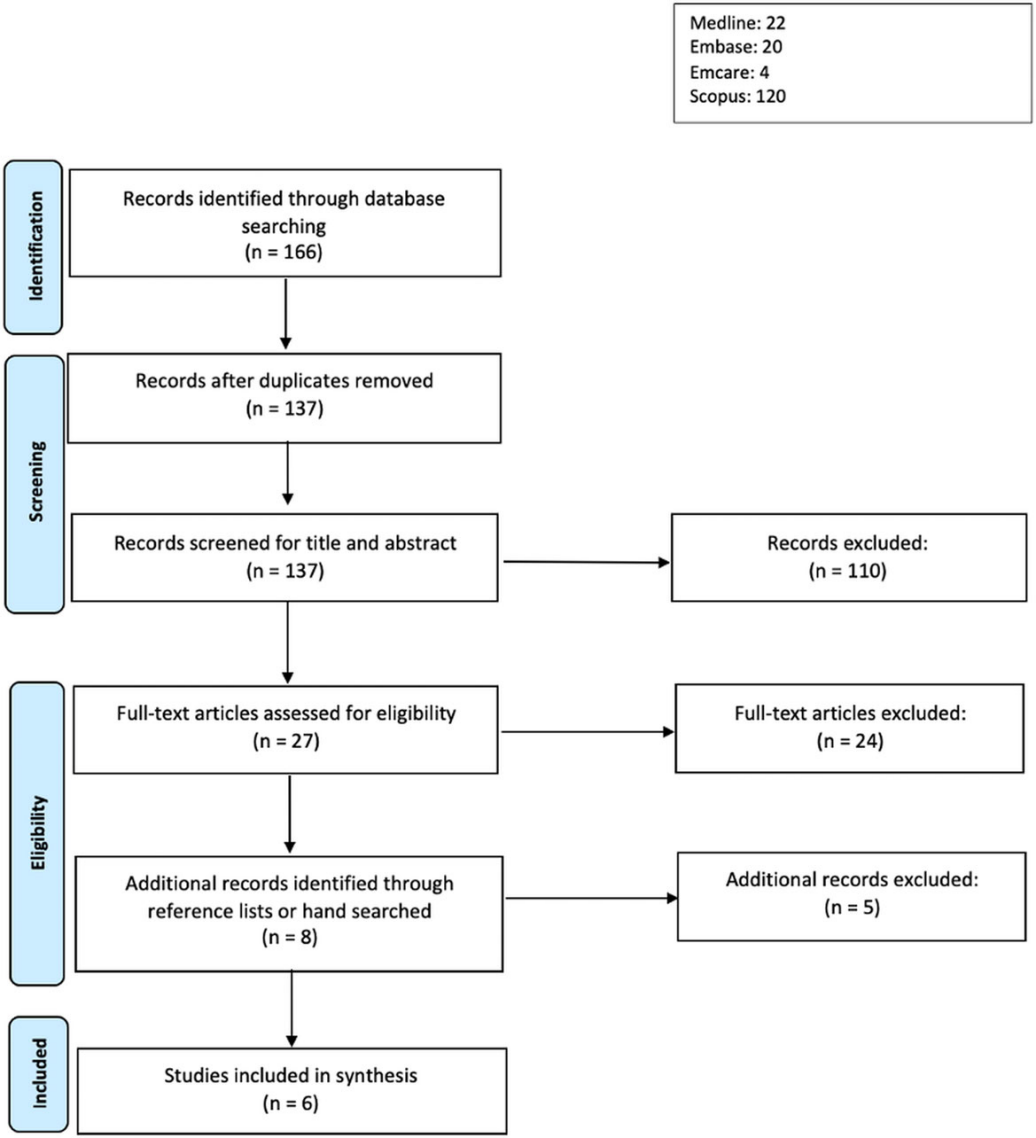
PRISMA flow diagram of study selection.

The initial search strategy was applied to papers in the last twenty years, in accordance with common search conventions. The restriction was then expanded to 1980 to include more papers. Following searching of primary sources, articles were then imported into Covidence, a data screening tool, for further screening and analysis. Duplicates were then removed and articles were then screened by title and abstract for relevance. Articles that passed title and abstract screening were then screened by full text before inclusion (Figure 1). Title and abstract screening of the articles were done by two independent reviewers, CC and QS, to ensure that each article was double screened.

Full text screening was then undertaken for articles included after title and abstract screening. Each full text assessment was also undertaken by two independent reviewers, BD and QS, to ensure that each included article was doubly assessed as a full text.

Data extraction collected relevant information on the (1) key study characteristics (for example, publication year, country, income classification of the country etc.), (2) key findings of each paper, (3) bacterial counts in the environment and/or surfaces as recorded in each paper, and (4) clinically significant events and/or clinically significant infections as recorded in each paper.

For the purposes of this review, narrative synthesis was employed to qualitatively describe the data extracted.

## Results

166 papers were initially included for screening. After duplicates were removed, 137 studies were left (Figure 1). The 137 studies were then screened by title and abstract for relevance, with 27 studies remaining after screening (Figure 1). The 27 studies were finally assessed as full texts for eligibility, and after four additional articles were identified from reference lists and included, six studies were included in the final review. Of the six papers^13–18^ that were included in this review, all were from high income countries (Table 2). Two of these papers^14,16^ were from the United Kingdom and four^13,15,17,18^ were from the United States. In terms of study design, 50% (*n* = 3) of the included papers were quasi-experimental studies, while the other 50% (*n* = 3) were cross-sectional studies.

**Table 2.** Characteristics of included papers, key findings, bacterial counts, and clinically significant events/infections

Author, Year, Country	Income Classification of Country	Study Design	Participants	Key Findings	Bacterial counts in the environment and/or surfaces
Alexander^13^, 2013, United States	High	Cross-sectional microbial surveillance study	33	Swabs from shoe covers had lower bacterial growth than swabs from uncovered shoes	Swabs from uncovered shoes had 60 CFUs compared to 20 CFUs on swabs from shoe covers (*P* = .01)
Carter^14^, 1990, United Kingdom	High	Cross-sectional Study	131	Higher CFUs in area where shoe covers were worn (Clean area) compared to areas where shoe covers were not (Dirty area). Bacteria were found in swabs from hands after application of overshoes more often than in swabs from hands prior to application of overshoes.	Floor CFU counts trial 1: “Clean” area: 23 “Dirty” area: 17 Floor CFU counts trial 2: Clean” area: 462 Dirty” area: 50 Bacterial growth on hands: Before overshoe application – 20% After overshoe application – 100%
Copp^15^, 1987, United States	High	Quasi-experimental study	51	The use of shoe covers resulted in lower CFU on swabs from OR floors compared to the wearing of uncovered street shoes	Swabs from OR floors when street shoes worn 35.7 CFU Swabs from OR floors when shoe covers worn 7.5 CFU (*p*-value = .001)
Humphreys^16^, 1991, United Kingdom	High	Quasi-experimental study	40	The wearing of shoe covers did not alter the CFU on swabs from operating room floors	Theater floor CFU count with shoe covers: 64.37 Theater floor CFU count without shoe covers: 67.51 (*p*-value = .5)
Malhotra^17^, 2022, United States	High	Cross-sectional comparative study	4,367	Reduced PPE use not linked to higher SSIs, supports re-evaluation of routine disposable PPE policies	Surgical site infections preintervention = 5.1% Surgical site infections postintervention = 2.6% (*p*-value = .001)
Ritter^18^, 1984, United States	High	Quasi-experimental study	10	Swabs from shoe covers had lower bacterial growth than swabs from uncovered shoes	Swabs from uncovered shoes had 60 CFUs compared to 20 CFUs on swabs from shoe covers (*p*-value = .01)

*Note.* CFU, colony forming units, OR, operating room.

### Summary of clinically significant events

One paper (Malhotra *et al.*) discussed clinically significant events in the context of OR shoe cover use.^17^ The researchers examined the impact of the elimination of disposable OR attire such as shoe covers, disposable head covers and single-use face masks on SSI rates at their institution. These changes in infection prevention measures were associated with a significant decrease in SSIs from 5.1% to 2.6% (*P* < .001) even though the postintervention period was accompanied by an increase in surgical volume and in the proportion of contaminated and dirty cases. Although this study did not examine the use of shoe covers in isolation, the elimination of shoe cover use was associated with a reduced rate of SSIs. No data were found in the other five papers on clinically significant events and/or infections.

### Summary of bacterial counts data

Five papers reported on bacterial counts in the OR environment and/or on surfaces^13 14 15 16 18^ (Table 3).

**Table 3.** Summary of CFU findings’

Author, Year, Country	CFU without shoe covers	CFU with shoe covers	Absolute (% change)	Direction of effect
Alexander^13^, 2013, United States	60	20	−40 CFU (−67%)	Favors shoe covers
Carter^14^, 1990, United Kingdom	17–50	23–462	Increase in CFU	Favors no shoe covers / potential harm
Copp^15^, 1987, United States	35.7	7.5	−28.2 CFU (−79%)	Favors shoe covers
Humphreys^16^, 1991, United Kingdom	67.51	64.37	−3.14 CFU (−4.6%)	No effect
Ritter^18^, 1984, United States	60	20	−40 CFU (−67%)	Favors shoe covers

Overall, two papers described a fall in bacterial counts associated with wearing shoe covers. Alexander *et al*. found that bacterial colony counts on shoe covers were significantly lower than on uncovered shoes. (*P* = .01)^13^ Similarly, Copp *et al*. also showed that shoe covers transferred fewer bacteria onto the study area than street shoes without shoe covers.^15^ However, two included papers showed no association between bacterial count and the use of shoe covers. Humphreys *et al*. showed that use of shoe covers was not associated with a decrease in the mean OR floor bacterial colony counts (67.51 without shoe covers compared with 64.37 with shoe covers. [*P* = .5])^16^ Ritter *et al*. similarly showed no significant difference in mean colony forming units on swabs from OR floors when shoe covers were worn compared to when shoe covers were not worn.^18^ Finally, Carter *et al*. actually found that wearing shoe covers was associated with higher operating room floor CFUs than not wearing shoe covers.^14^ They also found an association between donning/doffing of shoe covers and increased bacterial counts on the hands of those wearing the shoe covers.

## Discussion

Major professional bodies recommend shoe covers only for splash or fluid exposure, not for infection prevention.^11,12,13^ However, if shoe covers are to fulfill their role as a barrier against contamination, maintaining structural integrity and remaining intact should be a key function. Jones *et al*. found that 12% of unused shoe covers tested had holes in them, while 70.2% of used shoe covers had holes in them, eventually concluding that shoe covers are ineffective as a “barrier” method.^19^ The absence of clinical SSI benefit in this review demonstrates that routine shoe cover mandates for infection control exceed the available evidence, indicating that current practice is misaligned with both data and guideline intent.

From an infection prevention perspective, the premise that shoe covers reduce SSIs and/or lead to a reduction in OR bacterial floor counts remains unsubstantiated.^20^ In the studies included in this review, only one paper by Malhotra *et al*. investigated a link between shoe covers and clinically significant consequences, establishing that a decreased use of shoe covers was associated with a significant decrease in SSIs.^17^ Although no validated causal relationship exists between OR floor CFU counts and SSI rates, the findings do support the lack of a positive association between shoe cover use and a decrease in SSIs. This further underscores that SSIs are driven primarily by factors other than floor contamination, conceivably patient factors, surgical technique, antimicrobial prophylaxis etc.

Furthermore, the review highlighted the possibility of disposable shoe coverings acting as a potential avenue of contamination. This finding was demonstrated in the study by Carter *et al*., showing an increase in bacterial contamination of the hands of HCW who had just removed or put on shoe covers compared to swabs taken before they did so.^14^ Although this theoretically should not directly increase the risk of SSIs if HCW perform appropriate surgical hand antisepsis, increasing the bacterial load of hands demonstrates a plausible mechanism of cross-contamination.^21^ However, no other studies examined this risk, and further investigation into the validity of these findings is required.

In terms of data on how shoe covers impact OR floor bacterial counts, the published literature was conflicting. Although two papers in our review described an association of lower bacterial counts with use of shoe coverings, two other papers found no difference and the fifth paper found higher CFUs on OR floors where shoe covers were used. These findings are similar to those in Eisen’s 2011 review.^20^

In light of the evidence which fails to identify a benefit of shoe covers in preventing SSIs and conflicting evidence on their ability to reduce contamination of the OR as well as the lack of biological plausibility, the question of their utility in preventing SSIs should be re-examined, particularly with the environmental benefits and financial savings reducing their use may produce.

### Limitations and future research

The sample size of six papers within this review was relatively small (ranging from 10 to 1,463 participants), which is indicative of the scant literature available on this topic. Many of the included papers were published quite some time ago, with only two being published in the past twenty years. Furthermore, the studies have small sample sizes and cross-sectional and quasi-experimental designs with a high risk of bias due to lack of randomization, uncontrolled confounding, and inconsistent outcome measurement, which limits causal inference. Additionally, most studies failed to measure infection outcomes directly, making it difficult to assess the clinical relevance of the changes in CFU counts. Language bias was also present as only English language papers were included.

We therefore recommend limiting the use of shoe covers to situations where HCWs or visiting individuals are at risk of gross contamination with blood or body fluids and endorsing the use of dedicated OR shoes in restricted and semi-restricted areas, in alignment with the ACORN and AORN guidelines.^11,12^ For regular OR HCW, it would be appropriate to require dedicated footwear that is worn exclusively within the OR. The only scenario in which shoe covers may offer some benefit is when individuals enter the OR wearing street shoes, where there may be a risk of environmental contamination from gross soil on the shoes, though our review found no evidence to support this concern. Additionally, regular environmental cleaning and minimizing traffic in and out of the OR can further help to reduce the risk of contamination.

Nonetheless, the evidence we have collected suggests that there is no strong evidence to oppose phasing out the routine use of disposable shoe covers. Any position suggesting otherwise should be required to provide evidence that the use of shoe covers has clinically significant patient benefits and, while this could be the subject of future research, should not delay the phasing out of shoe covers.

This review demonstrates that there is little evidence supporting the use of disposable shoe covers in ORs as continued use of shoe covers comes at both an environmental and financial cost to healthcare organizations.

## Supporting information

Soh et al. supplementary material Soh et al. supplementary material

## Supplementary material

The supplementary material for this article can be found at https://doi.org/10.1017/ash.2026.10311.
