# Supplementary material for: Does the evidence support the use of operating room shoe covers to prevent surgical site infections? A scoping review
Source: Antimicrob Steward Healthc Epidemiol. 2026 Feb 24;6(1):e54. doi: 10.1017/ash.2026.10311 (PMC12936802; doi:10.1017/ash.2026.10311)
Supplement: Soh et al. supplementary material [file S2732494X26103118sup001.docx]

**Supplementary Materials**

| **Database** | **Search Strategy** |
| --- | --- |
| Ovid Emcare | 1 Shoes/ 5216  2 shoe*.mp. [mp=title, abstract, heading word, drug trade name, original title, device manufacturer, drug manufacturer, device trade name, keyword heading word] 8336  3 1 or 2 8336  4 Operating Rooms/ 12634  5 ((surgery or surgical) adj2 (room* or theater* or theatre* or suite*)).mp. [mp=title, abstract, heading word, drug trade name, original title, device manufacturer, drug manufacturer, device trade name, keyword heading word] 1111  6 (operating adj2 (room* or theater* or theatre* or suite*)).ti,ab,kf. 19814  7 4 or 5 or 6 23853  8 exp Infection Control/ 27627  9 (infect* adj2 (control* or prevent*)).ti,ab,kf. 32504  10 8 or 9 51089  11 3 and 7 and 10 4  12 from 11 keep 1-4 4 |
| Embase Classic+Embase | 1 Shoes/ 11842  2 shoe*.mp. [mp=title, abstract, heading word, drug trade name, original title, device manufacturer, drug manufacturer, device trade name, keyword heading word, floating subheading word, candidate term word] 22303  3 1 or 2 22303  4 Operating Rooms/ 52589  5 ((surgery or surgical) adj2 (room* or theater* or theatre* or suite*)).mp. [mp=title, abstract, heading word, drug trade name, original title, device manufacturer, drug manufacturer, device trade name, keyword heading word, floating subheading word, candidate term word] 3808  6 (operating adj2 (room* or theater* or theatre* or suite*)).ti,ab,kf. 60858  7 4 or 5 or 6 82519  8 exp Infection Control/ 128403  9 (infect* adj2 (control* or prevent*)).ti,ab,kf. 116784  10 8 or 9 214387  11 3 and 7 and 10 20 |
| Ovid MEDLINE(R) ALL | 1 Shoes/ 7135  2 shoe*.mp. [mp=title, book title, abstract, original title, name of substance word, subject heading word, floating sub-heading word, keyword heading word, organism supplementary concept word, protocol supplementary concept word, rare disease supplementary concept word, unique identifier, synonyms, population supplementary concept word, anatomy supplementary concept word] 14214  3 1 or 2 14214  4 Operating Rooms/ 16037  5 ((surgery or surgical) adj2 (room* or theater* or theatre* or suite*)).mp. [mp=title, book title, abstract, original title, name of substance word, subject heading word, floating sub-heading word, keyword heading word, organism supplementary concept word, protocol supplementary concept word, rare disease supplementary concept word, unique identifier, synonyms, population supplementary concept word, anatomy supplementary concept word] 2452  6 (operating adj2 (room* or theater* or theatre* or suite*)).ti,ab,kf. 42146  7 4 or 5 or 6 50133  8 exp Infection Control/ 71060  9 (infect* adj2 (control* or prevent*)).ti,ab,kf. 90728  10 8 or 9 149747  11 3 and 7 and 10 22 |
| SCOPUS | ( TITLE-ABS-KEY ( shoe* ) AND TITLE-ABS-KEY ( ( operating AND room* ) OR ( surgery OR surgical ) OR ( room* OR theater* OR theatre* OR suite* ) ) AND TITLE-ABS-KEY ( ( infect* AND ( control* OR prevent* ) ) OR infection AND control ) ) |

*Supplementary Table 1: Search Strategies*
